# AGING AT HOME IN BRAZIL: COMMUNITY RESEARCH FROM AN ARCHITECTURAL PERSPECTIVE

**DOI:** 10.1093/geroni/igae098.1282

**Published:** 2024-12-31

**Authors:** Mariana Alves Silva do da Nascimento

**Affiliations:** University of São Paulo, São Paulo, Sao Paulo, Brazil

## Abstract

Environmental Gerontology has been an essential aspect of aging studies worldwide for years. However, its integration into fields like Architecture and Urban Planning is still limited in Brazil. Professionals who design urban spaces must consider how their decisions impact people’s lives, especially the older population who often face challenges accessing the city’s amenities. This presentation shares insights from a qualitative study conducted in São Paulo, Brazil with community residents aged 56 to 74 (N=23; mean age=64; 73% women). Using the Collective Subject Discourse (CSD) analysis technique to investigate their perceptions, preferences, and expectations of aging in place, participants highlighted the importance of features such as sidewalks, green spaces, subway lines, and accessible shops and services, to support daily activities and enable aging in place. Participants also expressed a desire for a range of activities and amenities tailored to older adults, reducing their reliance on relatives to fulfill their social needs. Furthermore, 39% of participants expressed a sense of attachment desiring to age in their current homes, indicating that home environments should also be continuously adapted to the wishes and needs of older individuals. This study contributes to the field by highlighting the importance of interdisciplinary education and of collaboration among professionals in aging, architecture, and urban planning to ensure the inclusion of older adults in the creation of age-inclusive environments for all.

